# Ozone

**Published:** 1849-07

**Authors:** 


### OZONE.

Professor Schonbein, the discoverer of gun-cotton, has proposed this name for an imaginary principle in the atmosphere, which he supposes to be evolved by electricity and to be concerned in the production of certain diseases. In some experiments made a few years since, a peculiar odor was remarked when electricity passed from pointed bodies into the air, and he supposed a substance was produced at the time similar to that which becomes apparent at the positive electrode on the decomposition of water, by a voltaic pile, or by the action of phosphorus on moist air. Owing to its offensive odor it was styled *ozone*, from *ozo*, to stink. What the peculiar matter is which gives rise to this odor has not been determined. Schonbein regards it as a compound of hydrogen and oxygen, the latter element being in excess—a deutoxide or tritoxide of hydrogen; but no analytical evidence has been brought to the support of this hypothesis. Berzelius and De la Rive attribute the odor to the presence of oxygen, modified either by electricity or by the catalytic activity of other substances—"an allatropic modification of oxygen." The substance, whatever it was, seemed to be a powerfully oxidizing agent, and when inhaled produced irritation of the air-passages, and all the symptoms of catarrh. As electricity is continually acting on the air, Prof. Schonbein concluded that ozone must be continually evolved, and must be productive of catarrhal and pulmonary affections. It was ascertained that iodide of potassium was decomposed by the body liberated in the electrical experiments; that paper rubbed with the iodide and starch became blue when exposed to its action. It was also remarked by Schonbein and his friends that the same discoloration took place when paper, so prepared, was exposed to the air during the prevalence of influenza, and that the discoloration was most intense when influenza was at its height. Hence he concluded that ozone was the cause of influenza. This conjecture has been extended.

Influenza is frequently the precursor of cholera. Dr. Herrick and Dr. Bird, of Chicago, have inferred from this coincidence that they may have a common cause, and that this cause may be ozone. They think they have detected ozone in the atmosphere of Chicago since cholera made its appearance there.

Sulphur and its compounds decompose ozone, and therefore persons living near sulphur springs, or engaged in manufactures in which sulphurous acid is liberated, ought to escape the diseases to which it gives rise. Sulphur ought also to be a remedy for the diseases engendered by it. Such is the train of argument.

It would be difficult, we think, among the delusions of medicine, to find a better specimen of loose reasoning and hasty generalization than this ozone theory affords. We know not that such a substance exists. We are not sure that it is ever present in the air. If generated by electricity, there is no satisfactory proof that it is capable of producing the effects ascribed to it. If produced by electricity we cannot understand why its appearance should be periodical when the cause is continually operating. But supposing it to be established that it is the cause of influenza, a disease generally of cold weather, what probability is there that it is also the cause of cholera, a disease so different in character, and generally one of hot weather? Certainly few conjectures could be more absurd than that cholera and influenza are the products of the same poison. But conceding all this, is it true that cholera has avoided the vicinity of sulphur springs and manufactories? On the contrary, it was no where more fatal in Kentucky, in 1833, than in the neighborhood of some of its sulphur springs. And then as to the remedy. If sulphur decomposes ozone, does it hence follow that it will cure cholera? Allow that it may neutralize the poison, must it necessarily overcome the effects of the poison? But we need not push our objections further. The hypothesis is too flimsy to stand the slightest examination. Cholera, according to this crude notion, is to be cured by a few grains of sulphur, administered *pro re nata*.

